# Supplementary material for: A New Cell Model for Investigating Prion Strain Selection and Adaptation
Source: Viruses. 2019 Sep 22;11(10):888. doi: 10.3390/v11100888 (PMC6832381; doi:10.3390/v11100888)
Supplement: Supplementary file 1 [file viruses-11-00888-s001.pdf]

Supplementary Materials

Supplementary Table S1. Changes in the likelihood of isolating susceptible and refractory cells from generation 1 to generation 3.

1st generation (PK1 subclones)		
**Susceptibility type**	**Ratio (phenotypic/total cells)**	**Likelihood**
Me7 susceptible	1/720	$1.4 * 10^{-3}$
Me7 refractory	719/720	0.99
RML susceptible	717/720	0.99
RML refractory	3/720	$4.2 * 10^{-3}$
**2nd generation (PME2 subclones)**		
**Susceptibility type**	**Ratio (phenotypic/total cells)**	**Likelihood**
Me7 susceptible	26/960	$2.7 * 10^{-2}$
Me7 refractory	934/960	0.97
RML susceptible	886/960	0.92
RML refractory	74/960	$7.7 * 10^{-2}$
22L susceptible	688/960	0.72
22L refractory	272/960	0.28
**3rd generation (PME2-6D8 subclones)**		
**Susceptibility type**	**Ratio (phenotypic/total cells)**	**Likelihood**
Me7 susceptible	295/468	0.63
Me7 refractory	173/468	0.37
RML susceptible	468/468	1
RML refractory	0/468	0
22L susceptible	468/468	1
22L refractory	0/468	0

The number of phenotypic (susceptible or refractory to prion strains Me7, RML and 22L) clones are listed as ratio to the total number of cells isolated per generation. The likelihood of isolating such phenotypic cells (Likelihood) was calculated from the ratio of phenotypic/total number of cells isolated.

Supplementary Table S2. Infectious titers of embryonic cortico-hippocampal cultures from FVB wt ($Prnp^{+/+}$) and FVB ko ($Prnp^{-/-}$) mice, infected with RML, Me7, PME2-6D8 and uninfected CD1.

Primary cells	Inoculum	Prnp genotype	Days post infection	Log [TCIU]
**Primary Neuronal**	RML, 10^{-5}	wt	2 weeks	5.2
	RML, 10^{-5}	wt	3 weeks	5.6
	RML, 10^{-5}	*$Prnp^{-/-}$*	2 weeks	4.6
	RML, 10^{-5}	*$Prnp^{-/-}$*	3 weeks	4.6
**Primary Neuronal**	Me7, 10^{-5}	wt	2 weeks	2.1
	Me7, 10^{-5}	wt	3 weeks	<2.1
	Me7, 10^{-5}	*$Prnp^{-/-}$*	2 weeks	2.1
	Me7, 10^{-5}	*$Prnp^{-/-}$*	3 weeks	2.6
**Primary Neuronal**	PME2-6D8 [Me7], 10^{-5}	wt	2 weeks	5.2
	PME2-6D8 [Me7], 10^{-5}	wt	3 weeks	5.6

	PME2-6D8 [Me7], 10^{-5}	*Prnp*$^{-/-}$	2 weeks	5.3
	PME2-6D8 [Me7], 10^{-5}	*Prnp*$^{-/-}$	3 weeks	5.3
**Primary Neuronal**	RML, 10^{-4}	wt	2 weeks	5.7
	RML, 10^{-4}	wt	3 weeks	>6
	RML, 10^{-4}	*Prnp*$^{-/-}$	2 weeks	5.6
	RML, 10^{-4}	*Prnp*$^{-/-}$	3 weeks	5.8
**Primary Neuronal**	Uninfected CD1, 10^{-4}	wt	3 weeks	<2.1
	Uninfected CD1, 10^{-4}	*Prnp*$^{-/-}$	3 weeks	<2.1

Primary cortico-hippocampal cultures from FVB ko (Prnp$^{-/-}$) and wt (Prnp$^{+/+}$) mice were infected with inocula specified above and infectious titers determined with PME2-6D8 cells using the "Scrapie cell assay in end point format" (SCEPA) as specified in Materials and Methods. Infectious titres are expressed as Log [tissue culture infectious units] (Log [TCIU]s).

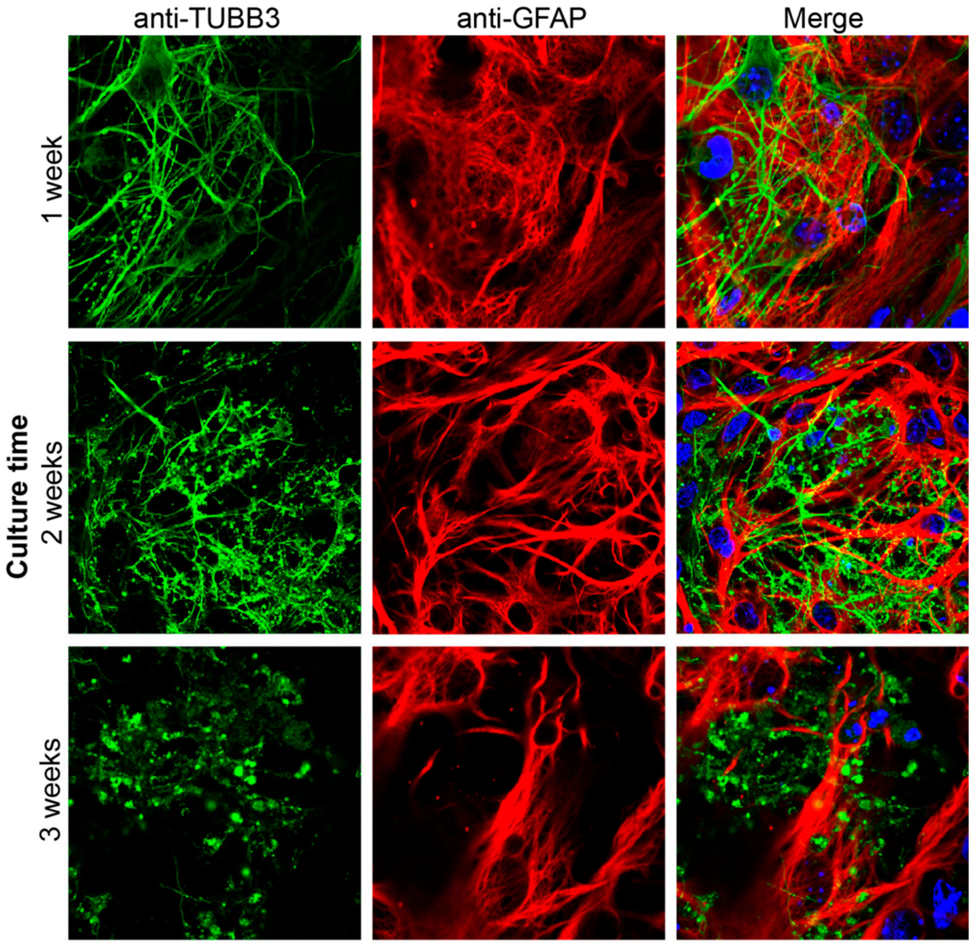

Supplementary Figure 1: Neuronal degeneration in mixed cortico-neuronal cultures. Primary cortico-hippocampal cultures from embryonic e17 FVB mouse brains were prepared as specified in Materials and Methods and plated onto poly-L-lysine-coated chamber slides in complete Neurobasal medium, supplemented with 2% (v/v) B27, 0.25% (v/v) GlutaMAX and 1% PS. The medium was changed twice weekly and cells were fixed by the end of weeks 1, 2 and 3. Representative images of mixed cortico-neuronal cultures, showing neuronal degeneration are shown.
